# Increased Expression of Beta-Defensin 1 (DEFB1) in Chronic Obstructive Pulmonary Disease

**DOI:** 10.1371/journal.pone.0021898

**Published:** 2011-07-19

**Authors:** Ellen Andresen, Gunar Günther, Jörn Bullwinkel, Christoph Lange, Holger Heine

**Affiliations:** 1 Section of Immunoregulation, Division of Innate Immunity, Research Center Borstel, Leibniz-Center for Medicine and Biosciences, Borstel, Germany; 2 Division of Clinical Infection Diseases, Department of Pneumology, Research Center Borstel, Leibniz-Center for Medicine and Biosciences, Borstel, Germany; 3 Division of Immunoepigenetics, Department of Immunology and Cell Biology, Research Center Borstel, Leibniz-Center for Medicine and Biosciences, Borstel, Germany; University Hospital Freiburg, Germany

## Abstract

On-going airway inflammation is characteristic for the pathophysiology of chronic obstructive pulmonary disease (COPD). However, the key factors determining the decrease in lung function, an important clinical parameter of COPD, are not clear. Genome-wide linkage analyses provide evidence for significant linkage to airway obstruction susceptibility loci on chromosome 8p23, the location of the human defensin gene cluster. Moreover, a genetic variation in the defensin beta 1 (DEFB1) gene was found to be associated with COPD. Therefore, we hypothesized that DEFB1 is differently regulated and expressed in human lungs during COPD progression. Gene expression of DEFB1 was assessed in bronchial epithelium and BAL fluid cells of healthy controls and patients with COPD and using bisulfite sequencing and ChIP analysis, the epigenetic control of DEFB1 mRNA expression was investigated. We can demonstrate that DEFB1 mRNA expression was significantly increased in bronchopulmonary specimen of patients with COPD (n = 34) vs. healthy controls (n = 10) (p<0.0001). Furthermore, a significant correlation could be detected between DEFB1 and functional parameters such as FEV_1_ (p = 0.0024) and the FEV_1_/VC ratio (p = 0.0005). Upregulation of DEFB1 mRNA was paralleled by changes in HDAC1-3, HDAC5 and HDAC8 mRNA expression. Whereas bisulfite sequencing revealed no differences in the methylation state of DEFB1 promoter between patients with COPD and controls, ChIP analysis showed that enhanced DEFB1 mRNA expression was associated with the establishment of an active histone code. Thus, expression of human DEFB1 is upregulated and related to the decrease in pulmonary function in patients with COPD.

## Introduction

Chronic obstructive pulmonary disease (COPD) is a complex inflammatory disorder influenced by environmental factors (especially cigarette smoking) and multiple genes. COPD is a leading cause of morbidity and mortality worldwide [1]. The clinical course of patients with COPD is closely related to the progression of pulmonary inflammation and the decline in lung function, which is an important clinical parameter of the disease. Genome-wide linkage analyses in the Boston Early-Onset COPD study have provided significant evidence for linkage of airway obstruction to chromosome 8 [2] and, in particular, for forced expiratory volume in 1 second (FEV1) to the region 23 of chromosome 8p [3]. Interestingly, the major human beta-defensin gene cluster, including human beta defensin 1 (DEFB1), maps to the same region [4]. Moreover, a genetic variation identified in exon 2 of *DEFB1* was found to be associated with COPD [5]. DEFB1 is an essential component of the human epithelia against invading pathogens and acts as an effector molecule of the host innate defense in the lung [6], [7]. However, little is known about specific components of the innate immune system that play a role in the progression of COPD. We hypothesized that DEFB1 is differently regulated and expressed in the lung during progression of COPD.

*DEFB1* maps to the chromosome 8p23.2-p23.1 and consists of two relatively small exons and an intron [8]. The first 128-bp exon encodes the signal sequence und propiece peptide; the second 234-bp exon encodes the mature peptide. DEFB1 is expressed constitutively in the airway epithelia and is not induced by inflammatory mediators or bacterial products [9]. It is able to kill or inactivate Gram-negative bacteria and fungi at micromolar concentrations through direct contact with microorganisms or indirectly by triggering innate and adaptive immune responses [10], [11]. DEFB1 is chemoattractive for immature dendritic cells and memory T-cells through the CCR6 chemokine receptor activation [12]. Because DEFB1 is believed to act as a baseline defense molecule in the absence of infection and inflammation, we focused on the role of epigenetic changes in regulation of DEFB1 gene expression. These changes include the DNA methylation state or modifications of the core histones, such as methylation and acetylation of specific residues of their N-terminal tails, which directly affect gene transcription [13], [14], [15]. A positive correlation has also been published between the disease severity and the reduction in histone deacetylase (HDAC) activity in the peripheral lung tissue of COPD patients [16]. Finally, the importance of DEFB1 in both, innate and adaptive immunity as well as its location on chromosome 8p23, where a significant evidence of linkage to COPD-related traits has been reported, makes DEFB1 an interesting candidate for the association with COPD progression.

## Methods

### Ethics statement

This study was approved by the Ethic Committee of the Medical Faculty of the University of Lübeck; Germany. All study participants provided written informed consent for the collection of bronchial epithelial cell biopsies and bronchoalveolar lavage (BAL) fluids.

### Patients

Healthy volunteer controls and patients with COPD with GOLD [1] stages 1–4 were recruited at the Medical Clinic of the Research Center Borstel, Germany. Following written informed consent bronchoscopy was performed according to national guidelines [17] obtaining epithelial cell biopsies from the middle lobe carina and BAL fluid with 200–300 ml of normal saline.

BAL fluid and biopsy specimens were collected from ten healthy subjects, two patients with mild COPD (stage 1), 13 patients with moderate COPD (stage 2), 15 patients with severe COPD (stage 3) and four patients with very severe COPD (stage 4). Healthy subjects for bronchoscopy were volunteers with FEV_1_/VC >70% of the predicted value and no signs or symptoms of COPD recruited by advertisement. Chronic illnesses (e.g. diabetes mellitus, renal insufficiency, HIV-infection) were exclusion criteria for study participant. Bodyplethysmography was performed using Jaeger MasterScreen 601045. The 6-min walking test (6MWD), arterial blood gas analysis, assessment of the MRC dypnoea scale and calculation of probability of survival based on BODE score [18] completed the clinical investigations. Acute exacerbations of COPD were defined according to the Anthonisen criteria [19].

### BAL fluid and bronchial epithelial cell biopsies preparation

BAL fluid cells were centrifuged (at 500 xg for 10 min, 4°C) and washed twice with PBS (at 500 xg for 5 min, 4°C). Cell viability was assessed with the use of the trypan-blue exclusion method. Bronchial epithelial cell biopsy specimens (two pieces from each study participant) were homogenized with the use of the Pellet Pestle Cordless Motor (Kontes, New Jersey, USA).

### Real-time quantitative PCR

Total RNA was isolated with the Absolutely RNA kit (Stratagene, La Jolla, CA, USA). Reverse transcription was performed in the presence of Superscript III Reverse Transcriptase (Invitrogen, Carlsbad, CA, USA). Gene transcript levels of defensin, beta 1 (DEFB1) and housekeeping gene β_2_-microglobulin (β_2_-M) were quantified by Real-time PCR with the use of LightCycler Fast Start DNA MasterPlus SYBR Green Master on a LightCycler 2.0 Instrument (Roche Applied Science, Mannheim, Germany) according to the manufacturer's instructions. Standard curves were obtained for each primer set with serial dilutions of plasmid DNA containing the amplification product. Absolute transcript levels are shown per one transcript of β_2_-M or as a common logarithm of this ratio. Sequences of the used primer sets were: DEFB1: sense 5′-TGTCTGAGATGGCCTCAGGT-3′, antisense 5′-GGGCAGGCAGAATAGAGACA-3′; β_2_-M: sense 5′-GCTGTGCTCGCGCTACTCTC-3′, antisense 5′-GCGGCATCTTCAAACCTCCAT-3′. Gene transcript levels of histone deacetylases (HDACs) 1-11 were quantified by Real-time PCR with the use of LightCycler 480 Probes Master and the universal probes #5 (HDAC2), #3 (HDAC3) and #58 (HDAC6) or LightCycler 480 SYBR Green I Master (HDAC1, 4, 5, 7–11) on a LightCycler 480 Instrument (Roche Applied Science, Mannheim, Germany). Variations of transcript levels between different samples were corrected by β_2_-M expression. Sequences of the primer sets used were: HDAC1: sense 5′-CCAAGTACCACAGCGATGAC-3′, antisense 5′-TGGACAGTCCTCACCAACG-3′; HDAC2: sense 5′- TGAAGGAGAAGGAGGTCGAA-3′, antisense 5′- GGATTTATCTTCTTCCTTAACGTCTG -3′; HDAC3: sense 5′-CACCATGCCAAGAAGTTTGA-3′, antisense 5′-CCCGAGGGTGGTACTTGAG-3′; HDAC4: sense 5′-GTGGTAGAGCTGGTCTTCAAGG-3′, antisense 5′-GACCACAGCAAAGCCATTC-3′; HDAC5: sense 5′-TCTGAATACCACACCCTGCTC-3′, antisense 5′-ACAAGGCAGCACAGCATACA-3′; HDAC6: sense 5′-AGTTCACCTTCGACCAGGAC-3′, antisense 5′-GCCAGAACCTACCCTGCTC-3′; HDAC7: sense 5′-CATGACCTCACAGCCATCTG-3′, antisense 5′-CATTGAGGTTGGGGTTTCTG-3′; HDAC8: sense 5′-CAGGATGGCATACAAGATGAAA-3′, antisense 5′-ATGGGATCCCCAGCTATTGT-3′; HDAC9: sense 5′-ATCCCAAGCTCTGGTACACG-3′, antisense 5′-TCTTGTGCTCCTGGTAATGTGT-3′; HDAC10: sense 5′-CTGTCAACCTGCCCTGGA-3′, antisense 5′-CAGCTCAGGGTCAAACTCAA-3′; HDAC11: sense 5′-TGTCTACAACCGCCACATCT-3′, antisense 5′-TGTTCCTCTCCACCTTATCCA-3′.

### Chromatin immunoprecipitation assay (ChIP)

Approximately 1.5×10^7^ BAL fluid cells from COPD patients and healthy controls at different clinical stages were washed twice with PBS and cross-linked for 10 min with 1% formaldehyde in serum-free medium at 37°C. Cross-linking was stopped with 1.25 M glycine. The following steps were carried out as previously described [20]. The following antibodies were used: 10 µl of polyclonal rabbit anti-dimethyl-Histone H3 (Lys9) and 10 µg of rabbit monoclonal Anti-trimethyl-Histone H3 (Lys4) (#07-712 and #05-745, respectively, Millipore (Billerica, MA, USA); 10 µl of polyclonal rabbit Anti-trimethyl-Histone H3 (Lys9) and Anti-trimethyl-Histone H4 (Lys20). Negative control was included omitting the antibody. The primer sets used for amplification were: DEFB1 (region I): sense 5′-CCACACTGGGGGCTCACTTTCT-3′, antisense 5′-TGGCCAAGGCTACCTTCTCA-3′; DEFB1 (region II): sense 5′-GAAGAGGGTGAAGTTTGAG-3′, antisense 5′-AGGCAGTTCACACTGGA-3′; DEFB1 (region III): sense 5′-AGAACTGCCTAACCTAGAAA-3′, antisense 5′-ACTTCTAATCGCTAACCCCT-3′; DEFB1 (region IV): sense 5′-GGTTAGCGATTAGAAGTTC-3′, antisense 5′-TGGAGCGTCACTGTATT-3′; DEFB1 (region V): sense 5′-GCAATCCACCAGTCTTAT-3′, antisense 5′-AGGCAACACTCAGGATT-3′; DEFB1 (region VI): sense 5′-GAGAACTTCCTACCTTCTGC-3′, antisense 5′-AGCCATCCGAGACTCAC-3′; DEFB1 (region VII): sense 5′-TGCCATAAGCACATTGC-3′, antisense 5′-GCCGAGAATAGCCAAGA-3′; DEFB1 (region VIII): sense 5′-CAATGTCTCTATTCTGCCT-3′, antisense 5′-TTCACTTCTGCGTCATT-3′; LINE-1: sense 5′-GCAGGCCTGGTGGTGAC-3′, antisense 5′-GCAGGCCTGGTGGTGAC-3′; Histone 4 : sense 5′-CATCACCAAGCCTGCCATTCGG-3′, antisense 5′-CACATCCATGGCTGTGACGGTC-3′. The fraction of immunoprecipitated DNA was determined by calculating of ratio of DNA present in the precipitates compared with the DNA in the input chromatin. Results were corrected by subtracting values of control reaction performed without antibodies and normalization on values of LINE-1 or Histone 4.

### Bisulfite sequencing analysis

Genomic DNA was purified from epithelial cell biopsy specimens obtained from five healthy controls und five COPD patients with the Easy-DNA Kit (Invitrogen, Carlsbad, CA, USA). 1 µg of genomic DNA was bisulfite converted using the EpiTect Bisulfite Kit (Qiagen, Hilden, Germany) according to manufacturer's instruction. Bisulfite-modified DNA was eluted with 20 µl of TE buffer and amplified with AccuPrime Taq DNA Polymerase High Fidelity (Invitrogen, Carlsbad, CA, USA) under the following conditions: 2 min at 94°C; followed by 35 cycles of 94°C for 30 sec, 55°C for 30 sec, 68°C for 60 sec; and a final extension of 68°C for 10 min. Bisulfite specific primers were designed by “BiSearch” (http://bisearch.enzim.hu/?m=search). Sequences of the primer sets used were: DEFB1 (Region 1, contained promoter region): sense 5′-TTTAGTGGGAAAAGAGAAAAGTT-3′, antisense 5′-AACTAATAAATTACACAACCTC-3′; DEFB1 (Region 2, contained exon 2 region): sense 5′-GGGTTAATTTTTTGGAAGAGAA-3′, antisense 5′-CACAAAATTCATTTTAACCC-3′. The PCR products were cloned into the pCR4-TOPO vector with the TOPO TA Cloning Kit for Sequencing using the TOP10 One Shot Chemically Competent cells (Invitrogen, Carlsbad, CA, USA) and 10–11 clones from each biopsy specimen were sequenced with T3 and T7 primer sets by GATC Biotech (Konstanz, Germany). Methylation analysis has been carried out with the MethTools software (http://genome.imb-jena.de/methtools/).

### Statistical analysis

Results are expressed as mean ± SD, mean ± SEM or median ±95% confidence interval. The one-tailed hypothesis was tested using unpaired t-test from log transformed data. Non-normal distributed values were tested with Mann-Whitney test. Analysis of variance of three or more unmatched groups was performed with the use of the One-way ANOVA test followed by Tukey's Multiple Comparison test or nonparametric Kruskal-Wallis test. When the results were significant, the unpaired t-test or Mann-Whitney test, respectively, was performed for comparison between groups. Correlation analyses were performed with the use of Pearson correlation in normal distributed values and Spearman nonparametric correlation. Statistics were performed with GraphPad Prism 5.02 software (GraphPad, San Diego, CA, USA) with differences *p<0.05, **p<0.01 and ***p<0.001 considered significant.

## Results

### DEFB1 mRNA expression is elevated in COPD and associated with functional lung parameters

The basic characteristics of the 34 patients with COPD with stages 1–4 and 10 healthy controls are shown in Table 1. Detailed characteristics of all study participants are summarized in supporting information, Table S1.

**Table 1:** Baseline characteristics of the study participants.

Characteristic	Healthy controls	COPD			
		Stage 1	Stage 2	Stage 3	Stage 4
**Number**	10	2	13	15	4
**Age [years]**	31.3±7.8	73.5±7.8	66.9±9.9	66.4±7.5	58.3±3.6
	(20–46)	(68–79)	(52–82)	(46–77)	(55–63)
**Sex [M/F]**	3/7	1/1	9/4	10/5	2/2
**BMI**	22.7±1.9	27.2±0.2	28.3–5.8	25.4±4.7	21.3±3.7
	(20.0–26.7)	(27.0–27.3)	(22.5–44.6)	(16.0–35.5)	(16.2–24.5)
**Pack years**	2.9±2.8	50.0±14.1	41.9±19.3	39.7±16.6	42.5±18.9
	(1–7)	(40–60)	(10–85)	(5–65)	(30–70)
**6MWD [m]**	516.0±49.3	305.0±169.7	352.3±127.1	291.0±122.3	367.5±88.6
	(430–625)	(185–425)	(150–545)	(0–465)	(240–430)
**FEV1 [%]**	104.2±16.6	89.9±2.0	63.9±10.2	38.2±6.2	26.1±2.8
	(60.7–117.4)	(88.5–91.3)	(50.1–79.4)	(30.2–47.3)	(22.8–29.0)
**VC [%]**	108.5–26.4	107.3±2.3	86.3±12.4	69.2±14.7	61.3±10.6
	(54.4–160.9)	(105.7–108.9)	(65.2–108.9)	(42.6–97.7)	(52.8–76.3)
**FEV1/VC [%]**	88.7±8.6	73.1±11.2	65.6±12.6	46.8±10.3	39.1±7.5
	(73.5–101.5)	(65.2–81.0)	(49.5–90.7)	(30.0–66.3)	(31.5–46.3)
**BODE score**	†	2.0±2.8	2.2±2.3	5.1±2.1	6.3±1.0
		(0.0–4.0)	(0.0–6.0)	(2.0–9.0)	(5.0–7.0)

Data are presented as mean±Std (range), COPD: chronic obstructive pulmonary disease, M: male, F: female, BMI: body mass index, pack years: the number of cigarettes smoked per day x number of years smoked)/20 (1 pack has 20 cigarettes), 6MWD: 6-minutes walking distance, FEV_1_: forced expiratory volume in one second, VC: vital capacity, BODE score: index, which incorporate body mass index, airflow obstruction, dyspnoea and exercise capacity ^18^, % of predicted value, † not determined. Stages 1 through 4 denote severity of disease according to the Deutsche Atemwegsliga and the Deutsche Gesellschaft für Pneumologie und Beatmungsmedizin ^27^, with higher number indicating greater severity. (Additional characteristics of all study participants are summarized in Table S1 in the supporting information).

**Figure 1:**
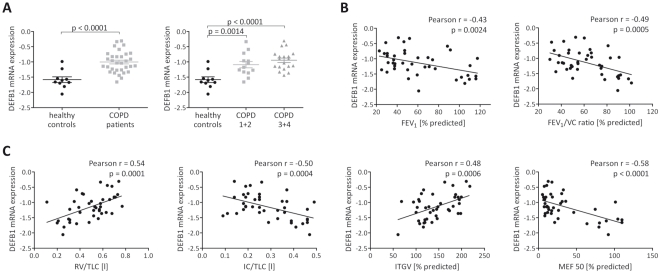
DEFB1 mRNA expression analysis in bronchial epithelial cell biopsies from patients with COPD and healthy controls. (**A**) Expression levels of DEFB1 mRNA in epithelial cell biopsies from patients with COPD (N = 34 total, N = 15 for COPD 1+2, N = 19 for COPD 3+4) and healthy controls (N = 10). Levels of DEFB1 mRNA were quantified by Real-time PCR, variations of transcript levels were corrected by β2-M mRNA levels and log transformed for statistical analysis. Differences between COPD patients (total) and healthy controls were tested using unpaired t-test. Analysis of variance of three groups was performed with the use of the One-way ANOVA test followed by Tukey's Multiple Comparison test. When the results were significant, the unpaired t-test was performed for comparison between the groups with differences p<0.05 considered significant. Results are presented as mean±SEM, each symbol represents a single sample. (**B**) Correlations between levels of DEFB1 mRNA and FEV_1_ and the ratio FEV_1_/VC and (**C**) correlations between levels of DEFB1 mRNA and the ratios RV/TLC, IC/TLC, ITGV and MEF50 in epithelial cell biopsies from patients with COPD (N = 34) and healthy controls (N = 10). Analyses in (B) and (C) were performed using Pearson correlations with differences p<0.05 considered significant. Each symbol represents a single sample. (The complete data and p values are given in supporting information, Table 2).

Absolute transcript levels of DEFB1 mRNA, normalized to the amount of β_2_-M, were higher in samples of bronchial epithelial cell biopsy from patients with COPD compared to the healthy controls (p<0.0001). There was a significant association between DEFB1 mRNA expression and samples with increasing severity of COPD (p = 0.0014 for patients with stage 1 and 2; p<0.0001 for patients with stage 3 and 4, Figure 1A). Furthermore, the increased DEFB1 mRNA expression significantly correlated with FEV_1_ (r = −0.43, p = 0.0024) and the FEV_1_/VC ratio (r = −0.49, p = 0.0005, Figure 1B). Significant correlation was also seen between DEFB1 mRNA and the ratios RV/TLC (r = 0.54, p = 0.0001) and IC/TLC (r = −0.50, p = 0.0004), ITGV (r = 0.48, p = 0.0006), MEF50 (r = −058, p<0.0001) and pAO2 (r = −0.50, p = 0.0004) when all subjects were included (Figure 1C). IC/TLC describes hyperinflation of the lung and is considered as an independent predictor of mortality [21]. However, there was no significant correlation of DEFB1 mRNA expression with pack years (r = 0.28, p = 0.1051), CRP level (r = 0.03, p = 0.8587) or BODE score (r = 0.05, p = 0.7727)(Table 2). By contrast, there was no difference in the level of DEFB4 mRNA among the groups of patients with COPD and healthy controls as the expression of DEFB4 mRNA was not correlated with increasing clinical severity of disease (p = 0.1716 for patients with stage 1 and 2; p = 0.2372 for patients with stage 3 and 4, compared to healthy controls; Supporting information, Figure S1). Furthermore, DEFB4 mRNA expression did not correlate with clinical parameters of COPD including airway obstruction, cigarette smoking and other lung function tests in the Pearson correlation analysis. These data are summarized in Table 2.

**Table 2:** Correlation analysis of DEFB1 and DEFB4 mRNA expression.

Parameter		DEFB1 mRNA expression		DEFB4 mRNA expression	
		Pearson r	P value	Pearson r	P value
FEV_1_	[l]	−0.52	**0.0002**	−0.20	0.2174
FEV_1_	[% predicted]	−0.43	**0.0024**	−0.12	0.4629
VC	[% predicted]	−0.29	**0.0338**	−0.27	0.0920
FEV_1_/VC	[% predicted]	−0.49	**0.0005**	0.00	0.9887
FEV_1_/FVC	[l]	−0.54	**0.0001**	−0.03	0.8784
IC/TLC	[l]	−0.50	**0.0004**	−0.20	0.2045
RV/TLC	[l]	0.54	**0.0001**	0.28	0.0774
ITGV	[% predicted]	0.48	**0.0006**	−0.04	0.7832
MEF50	[% predicted]	−0.58	**<0.0001**	−0.20	0.2097
pack years		0.28	0.1051	0.20	0.2449
BODE score		0.05	0.7727	0.20	0.2861
SGRQ		0.32	0.1760	−0.10	0.6995
CRP	[mg/dl]	0.03	0.8587	−0.04	0.8332
p_A_O_2_	[mm Hg]	−0.50	**0.0004**	−0.09	0.5775

DEFB1: defensin, beta 1, DEFB4: defensin, beta 4, FEV1: forced expiratory volume in one second, VC: vital capacity, FVC: forced vital capacity, IC: inspiratory capacity, RV: residual volume, TLC: total lung capacity, ITGV: intrathoracic gas volume, MEF50: maximal expiratory flow at 50%, pack years: the number of cigarettes smoked per day x number of years smoked)/20 (1 pack has 20 cigarettes), BODE score: index, which incorporates body mass index, FEV1, MRC dyspnoea scale and 6MWD ^18^, SGRQ: St George's Respiratory Questionnaire, CRP: c-reactive protein, paO2: arterial oxygen tension.

### HDAC1 mRNA level is elevated in COPD and correlates with DEFB1 mRNA expression

**Figure 2:**
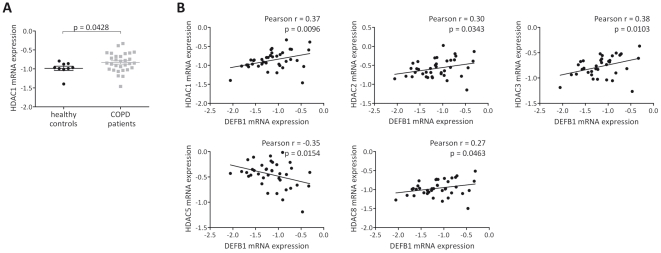
HDAC mRNA expression analysis in bronchial epithelial cell biopsies from patients with COPD and healthy controls. (**A**) Expression levels of HDAC1 mRNA in epithelial cell biopsies from patients with COPD (N = 34) and healthy controls (N = 10). Levels of HDAC1 mRNA were quantified by Real-time PCR, variations of transcript levels were corrected by β2-M mRNA levels and log transformed for statistical analysis. Analysis of variance between patients with COPD and healthy controls was performed by the use of the unpaired t-test with differences p<0.05 considered significant. Results are presented as mean±SEM, each symbol represents a single sample. (**B**) Correlations between levels of HDAC1 mRNA and HDAC1-3, 5 and 8 mRNA in epithelial cell biopsies from patients with COPD (N = 34) and healthy controls (N = 10). Analysis was performed using Pearson correlations with differences p<0.05 considered significant. Each symbol represents a single sample. (The complete data and p values are given in supporting information, Figures S2 and S3).

Since HDACs were also implicated in COPD [16], we next analysed whether the increased DEFB1 mRNA expression in patients with COPD was associated with the expression levels of the eleven human HDAC isoforms (HDAC1-11) in bronchial epithelial cell biopsies. The relative expression of HDAC1 mRNA normalized to β_2_-M was increased in samples from patients with COPD, as compared with samples from healthy controls (p = 0.0428) (Figure 2A). There were no significant differences between the mRNA expression of HDAC2-11 in samples from patients with COPD and those from healthy controls (supporting information, Figure S2A). Despite higher levels of HDAC1 mRNA in biopsies of patients with COPD, there was no significant correlation between the HDAC1 mRNA expression and the airway obstruction, as measured by the FEV_1_ (r = −0.13, p = 0.2185) and the FEV_1_/VC ratio (r = −0.20, p = 0.1118)(supporting information, Figure S2B). However, there was a positive correlation between the mRNA expression of DEFB1 and HDAC1-3 and 8 as well as a negative correlation between the DEFB1 and HDAC5 mRNA expressions when all subjects were included (Figure 2B).

### DNA methylation does not control DEFB1 mRNA expression level

**Figure 3:**
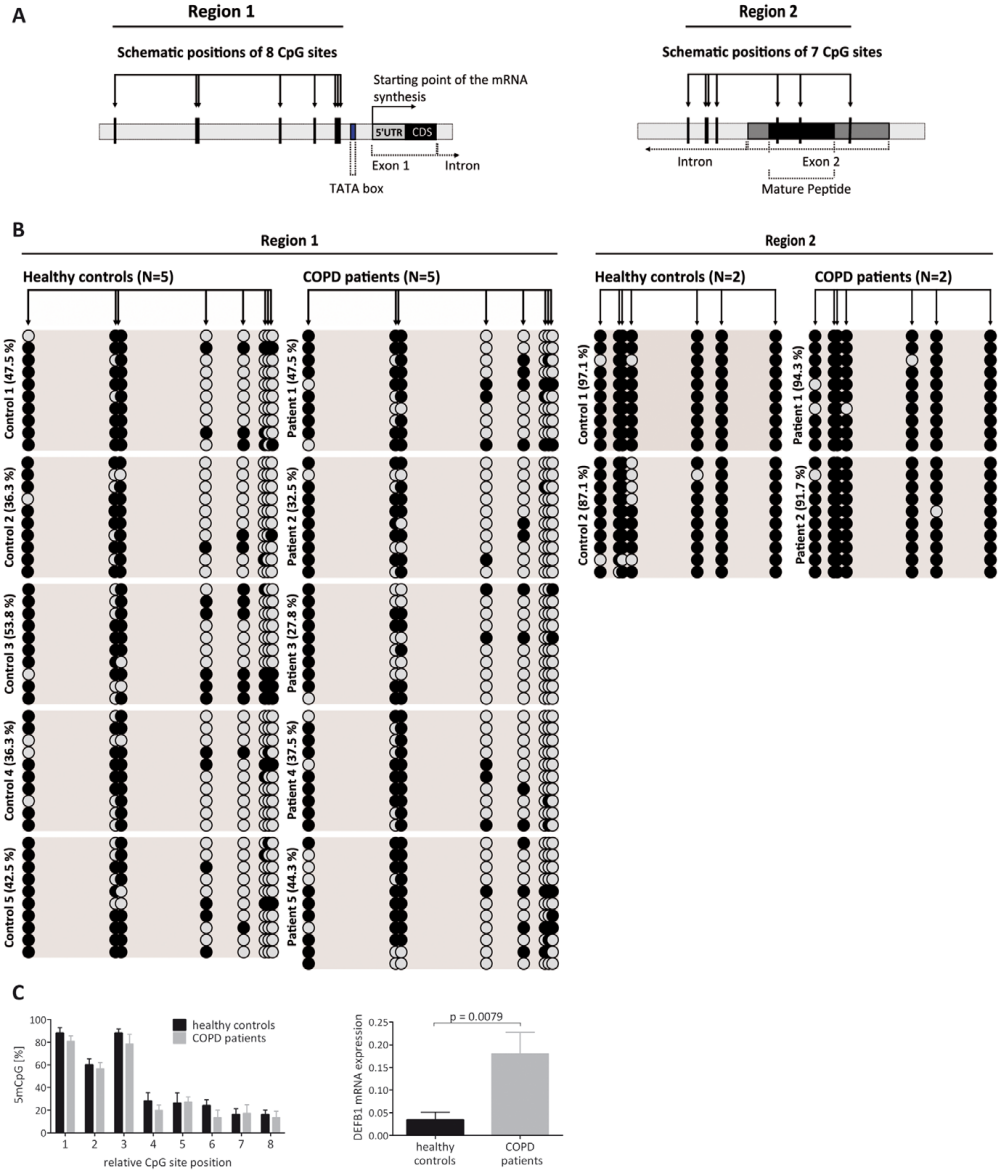
Bisulfite sequencing analysis of CpG methylation in bronchial epithelial cell biopsies. (**A**) Schematic view of the DEFB1 gene (NCBI GeneID: 1672) with exons displayed as filled boxes; the transcriptional start site is indicated; coding sequences are marked in black and 5′-UTR is shown in gray. Vertical arrows represent locations of eight CpG sites on the DEFB1 promoter (region 1) relative to the transcriptional start site and seven CpG sites on the DEFB1 downstream part of intron and exon 2 (region 2). (**B**) Bisulfite sequencing analysis of CpG methylation in bronchial epithelial cell biopsies of patients with COPD and healthy controls (both, N = 5 for region 1, N = 2 for region 2). Genomic DNA was treated as described, amplified by PCR and cloned for sequencing. At least 10 individual sequences (clones) were sequenced and analyzed per study subject. Each sequence (clone) is represented by a row and clones from the same subject are grouped. The individual CpG sites are arrayed in columns. Filled circles indicate methylated CpG; open circles indicate unmethylated CpG. (**C**) Average percentage of methylation for each CpG site of region 1 and levels of the corresponding DEFB1 mRNA expression for patients with COPD (N = 5, gray bars) and healthy controls (N = 5, black bars). Results are presented as mean±SEM.

To see whether DNA methylation plays a role in the regulation of DEFB1 transcription in bronchial biopsies of patients with COPD we compared the extent of methylation of individual CpG sites at the *DEFB1* locus to samples from healthy controls. Because the upstream part of the DEFB1 promoter (−700 bp) and whole DEFB1 gene sequence (+1 to +7324 bp) did not meet the criteria of a CpG island (length ≥200 bp, GC content ≥50% and obs_CpG_/exp_CpG_ ≥0.6) [22], the upstream part the DEFB1 promoter with eight CpG sites (region 1) and the downstream part of intron and exon 2 of DEFB1 with seven CpG sites (region 2) were analysed by sodium bisulfite sequencing (Figure 3A). At least 10 individual sequences (clones) were sequenced and analysed per patient with COPD and healthy control (Figure 3B). Each sequence (clone) is represented by a row and clones from the same subject are grouped. The individual CpG sites of the DEFB1 promoter and intron/exon 2 are arrayed in columns. Filled circles indicate methylated CpG sites, while open circles indicate unmethylated sites. Bisulfite sequencing revealed no differences in the magnitude of the methylation between the two groups at each CpG site despite the significant differences in the levels of DEFB1 mRNA (p = 0.0079) between samples from these two groups (Figure 3C).

### Extent of Histone H3 lysine 4 trimethylation correlates with DEFB1 mRNA expression level

**Figure 4:**
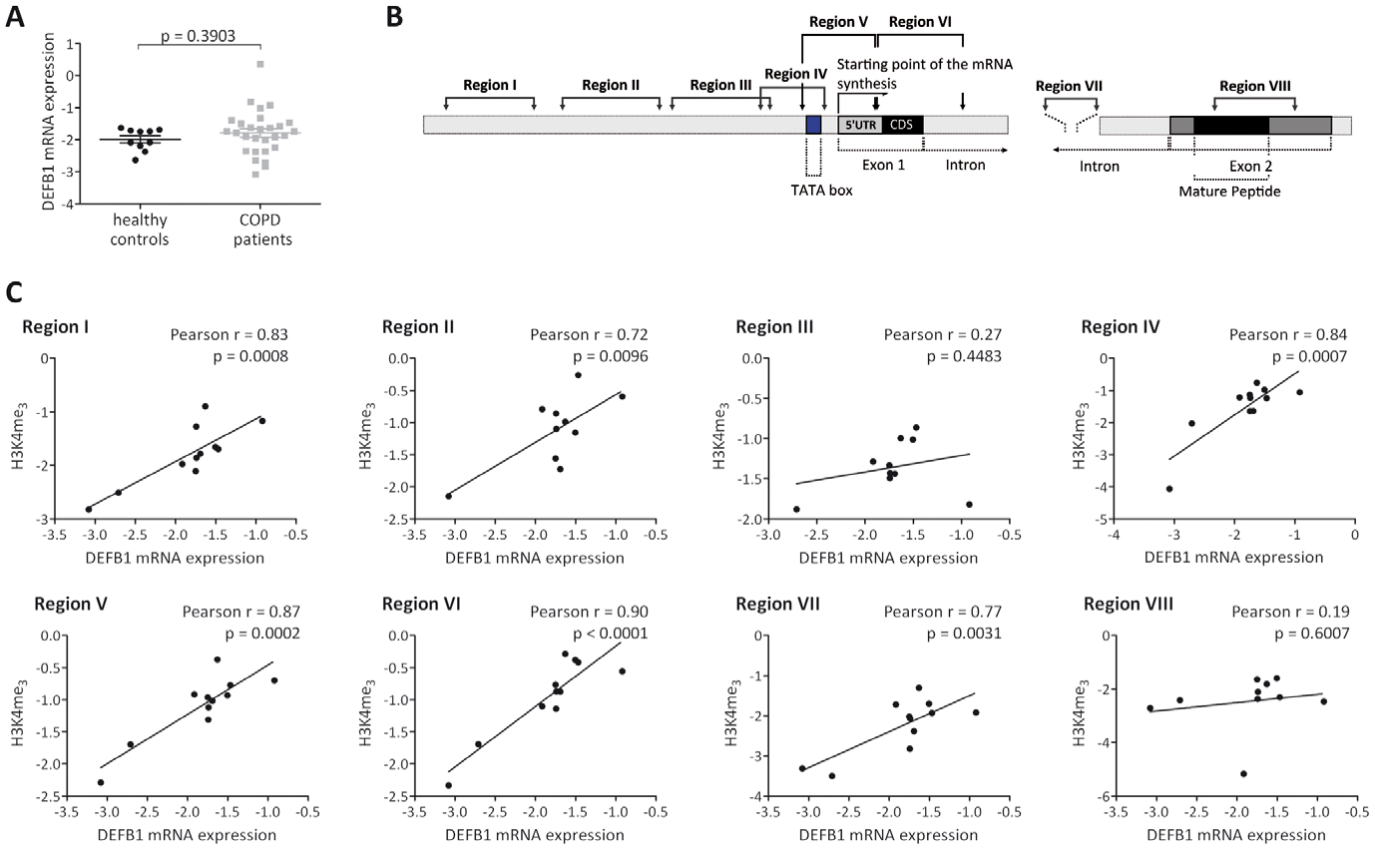
Analysis of histone H3 lysine 4 methylation (H3K4me_3_) within the DEFB1 locus in BAL fluid cells. (**A**) Expression levels of DEFB1 mRNA in BAL fluid cells from patients with COPD (N = 34) and healthy controls (N = 10). Levels of DEFB1 mRNA were quantified by Real-time PCR, variations of transcript levels were corrected by β2-M mRNA levels and log transformed for statistical analysis. Analysis of variance between patients with COPD and healthy controls was performed by the use of the unpaired t-test with differences p<0.05 considered significant. Results are presented as mean±SEM, each symbol represents a single sample. (**B**) Schematic view of the DEFB1 gene (NCBI GeneID: 1672) with exons displayed as filled boxes; the transcriptional start site and location of PCR primer sets used in ChIP experiments are indicated; coding sequences are marked in black and 5′-UTR is shown in gray. (**C**) Correlations between levels of H3K4me_3_ and DEFB1 mRNA in BAL fluid cells from study participants (N = 11) regardless of their COPD status. Levels of H3K4me_3_ were analyzed by ChIP using specific antibodies and quantified by Real-time PCR. The ChIP values are expressed as % of input corrected by subtracting values for no-antibody control and normalized on total histone H4. Correlations analysis was performed using Pearson correlations with differences p<0.05 considered significant. Each symbol represents a single sample.

The results described above showed that DEFB1 mRNA expression is upregulated in bronchial epithelial cell biopsies of patients with COPD and correlates with pathological changes characteristic for COPD. Since differences in the methylation state of the *DEFB1* promoter between patients with COPD and healthy controls were not detected, we wanted to analyze if other epigenetic marks were associated with the increased DEFB1 mRNA expression. Since material from bronchial epithelial cell biopsies was not sufficiently available to perform chromatin immunoprecipitation assays (ChIP), cells obtained from BAL fluid from patients with COPD and healthy controls were used. Levels of the DEFB1 mRNA in BAL fluid cells obtained from patients with COPD were slightly, but not significantly increased according to the analysis of variance (p = 0.3903), when compared with the levels in samples from healthy controls. However, there was a high degree of variation in DEFB1 mRNA expression for all subjects, in particular in patients with COPD (Figure 4A). Thus, the samples were used to analyze the histone modification pattern changes on the basis of the expression level of DEFB1 mRNA, but regardless of their COPD status. We selected four upstream regions located from −665 to −87 relative to the transcriptional start site (regions I to IV) and several regions throughout the DEFB1 loci from −123 to +7205 bp (regions V to VIII) (Figure 4B). Histone H3 lysine 4 trimethylation (H3K4me3) as an indicator for an active chromatin state was analysed in chromatin from BAL fluid cells by ChIP (N = 11) and then correlated with corresponding levels of the DEFB1 mRNA. With the exception of the promoter region III (r = 0.27, p = 0.4483) and region VIII within exon 2 (r = 0.19, p = 0.6007), the overall levels of H3K4me3 were significantly correlated with increasing levels of DEFB1 mRNA in the Pearson correlation analysis (region I: r = 0.83, p = 0.0008; region II: r = 0.72, p = 0.0096; region IV: r = 0.84, p = 0.0007; region V: r = 0.87, p = 0.0002; region VI: r = 0.90, p<0.0001; region VII: r = 0.77, p = 0.0031) (Figure 4C).

Altogether, these results corroborate our hypothesis that DEFB1 is associated with the progression of COPD and provide a first view on the epigenetic regulation of DEFB1 mRNA expression.

## Discussion

Currently, the role of the specific components of the innate immune system, especially defensins, and its clinical relevance in diverse clinical phenomena of COPD has not been extensively studied. COPD is a progressive inflammatory disease caused by the interaction of genetic susceptibility and environmental influences [23]. The human defensin beta 1(DEFB1) gene product, an endogenous antimicrobial peptide found in the airway epithelium [9], is believed to play an important role in mucosal immunity of the lung [24]. Moreover, genetic variations of DEFB1 identified in the 1654 G/A locus in the DEFB1 exon 2 coding for a valine to isoleucine substitution at position 38 (Val38Ile) and in the 668 C/G locus in 3′ flank of DEFB1 mRNA were found to be associated with COPD [5], [25]. The function of DEFB1 within the airway and its location on 8p, where evidence of linkage to qualitative and quantitative COPD-related phenotypes has been reported [2], [26], makes DEFB1 an interesting candidate for association with diagnosis of COPD and disease progression. Here we now demonstrate that the gene expression of DEFB1 is increased in samples of bronchial epithelial cell biopsy from patients with COPD, as compared with expression in healthy controls. Chronic airway obstruction, measured by reduction in forced expiratory volume in one second (FEV_1_) and the ratio of FEV_1_ to vital capacity or forced vital capacity (FEV_1_/VC and FEV_1_/FVC, respectively) [1], [27], is a cardinal feature of COPD. Their values are associated with disease severity and progression. For these quantitative phenotypes, our results provide an evidence for linkage to the DEFB1 gene expression in bronchial epithelial cell biopsies when all subjects were included. Interestingly, DEFB1 is located within the airway obstruction susceptibility loci on chromosome 8p23 with significant evidence of linkage to FEV_1_ with the highest LOD score of 3.30 [3]. In addition to FEV_1_, VC, FVC and the ratios FEV_1_/VC and FEV_1_/FVC, the analysis of other lung function tests assessed by bodyplethysmography or blood gas analysis gave similar results, suggesting that DEFB1 may be involved in the progression of airway obstruction. Since IC/TLC, describing hyperinflation of the lung, is considered as an independent predictor of mortality [21], DEFB1 gene expression may thus be identified as a novel genetic determinants of COPD-related phenotypes and a potential predictive marker for the outcome of COPD. However, despite the amplified support for genetic association with COPD, to our current state of understanding about the pathophysiology of COPD DEFB1 is not an obvious COPD susceptibility gene [28].

The DEFB1 gene (NCBI GenID: 1672) consists of two exons, encoding several forms of gene products ranging from 36 to 47 amino acid residues and differing from each other by amino terminal truncation [29]. For many years, the biochemical barrier function against invading pathogens by disrupting their cytoplasmic membrane and killing and/or inactivating particular spectra of bacteria and fungi was the major known role of DEFB1. Later evidence has shown that DEFB1 gene products constitutively produced by epithelial cells also display chemoattractant activity to immune cells [12], whose upregulation and secretion may amplify the immune response and contribute to airway inflammation seen in COPD. The ability of DEFB1 to promote necrotic and/or apoptotic cell death by increasing membrane permeability followed by the release of cytochrome c and activation of caspases [30], [31] suggests that DEFB1 could also be involved in the airway limitation and structural changes occurring in COPD. Finally, as disruption of the balance between apoptosis and regeneration of structural lung cells in the lung is reported to be important in the progressive destruction of healthy lung tissue during COPD [32], we speculate that DEFB1, the cytotoxic peptide abundantly produced by the airway epithelial cells of patients with COPD, may contribute to the apoptotic cell death within the conducting airway epithelium, an important upstream event in the pathogenesis of COPD.

With respect to the possible mechanisms involved in the regulation of DEFB1 in the airways of patients with COPD, the methylation analysis of CpG sites within the DEFB1 promoter and coding sequence showed no remarkable differences in the methylation pattern when bronchial epithelial cell biopsies obtained from patients with COPD were compared with biopsies from healthy controls. These results strongly argue against an important role of methylation of these CpG sites of the DEFB1 gene in the transcriptional regulation of DEFB1. Active histone modifications including histone H3 lysine 4 trimethylation (H3K4me3) are generally regarded as an indicator for active chromatin transcription and localized almost exclusively around the transcriptional start site [33], [34], [35]. Our ChIP experiments showed that H3K4me_3_ is present throughout the DEFB1 gene locus and, with exception of two positions within the promoter (region III) and exon 2 (region VIII), the levels of H3K4me3 correlate with transcriptional activity of *DEFB1*. Because H3K4me3 may well mark promoters and genes that became activated and provide the epigenetic setting to facilitate such activation [36], these results indicate that the establishment of such an active epigenetic code could be sufficient to establish the substantial induction of DEFB1 expression in the airways of COPD patients. Furthermore, histone H3 lysine 9 di- and trimethylation (H3K9me2 and H3K9me_3_) and histone H4 lysine 20 trimethylation (H4K20me_3_), which are generally correlated with transcriptional repression [37], [38], [39], [40], are present in promoter and coding regions of DEFB1. However, with the exception of the DEFB1 intron region VII and H3K9me_3_, the levels of these histone marks do not correlate with DEFB1 transcriptional activity. Since these marks can be observed in both, the repressive and active state [41], [42], [43], it seems likely that the combinatorial presence of such bipartite histone modifications may well represent the regulative event that seals transcriptional activation.

A possible scenario for DEFB1 gene activation in patients with COPD could also involve changes in histone deacetylases (HDACs) because the progressive reduction in both, the activity and expression of HDACs, especially HDAC2, in COPD has already been shown [16]. There are eleven classic human HDACs, HDAC1 to HDAC11, playing an essential role in gene regulation [44], [45]. In the present study we have shown that only the level of HDAC1 was increased in samples of bronchial epithelial cell biopsy from patients with COPD. In contrast to Ito et al. [16], we could not find any reduction in the levels of the HDAC2 mRNA previously reported in peripheral lung tissues and alveolar macrophages from patients with COPD. These discrepancies may be relatively specific to the different sample types (cells or tissues) used in our and others experiments. In addition, we found a positive correlation between levels of the HDAC1 and DEFB1 mRNA. Moreover, levels of the DEFB1 mRNA also positively correlated with expression of HDAC2, 3 and 8 mRNA and inversely correlated with expression of HDAC5 mRNA. These HDACs are also reported to be reduced in lung tissues and macrophages from patients with COPD in the above mentioned paper by Ito *et al*. Currently, we do not know if HDAC1 or the other HDACs directly positively affect DEFB1 mRNA expression. Although histone deacetylation is generally correlated with gene repression due to its condensing effect on chromatin structure, a number of publications point to the importance of rapid acetylation turnover at transcriptionally active sites [46], [47], [48]. Based on our results and because different HDACs appear to be involved in different cellular processes and presumable regulate different sets of genes [49], [50], we believe that these HDACs provide a molecular basis for the increased DEFB1 expression in the airways as COPD progresses.

Here we report that the expression of DEFB1 is increased in bronchial epithelial cell biopsies of patients with COPD and associated with pathological changes characteristic for COPD and disease severity. Shift of the epigenetic marks within the *DEFB1* gene locus and differentially expressed HDACs could have contributed to such gene activation events in COPD. Thus, further studies for DEFB1 and other molecules of the innate immune system may lead to the identification of novel genetic determinants in the development of COPD, which may lead to better understanding of COPD pathophysiology and new opportunities for prevention and treatment.

## Supporting Information

Figure S1 **DEFB4 mRNA expression in bronchial epithelial cell biopsies from patients with COPD and healthy controls.** Expression levels of DEFB4 mRNA in epithelial cell biopsies from patients with COPD (N = 34 total, N = 13 for COPD 1+2, N = 18 for COPD 3+4) and healthy controls (N = 10). Levels of DEFB4 mRNA were quantified by Real-time PCR, variations of transcript levels were corrected by β2-M mRNA levels and log transformed for statistical analysis. Differences between COPD patients (total) and healthy controls were tested using unpaired t-test. Analysis of variance of three groups was performed with the use of the One-way ANOVA test followed by Tukey's Multiple Comparison test. When the results were significant, the unpaired t-test was performed for comparison between the groups with differences p<0.05 considered significant. Results are presented as mean±SEM, each symbol represents a single sample.
(PDF)

Figure S2 **Histone deacetylase (HDAC) expression analysis in bronchial epithelial cell biopsies.** (**A**) Expression levels of HDAC1-11 mRNA in epithelial cell biopsies from COPD patients (N = 34) and healthy controls (N = 10). Levels of HDAC mRNA were quantified by Real-time PCR, variations of transcript levels were corrected by β_2_-M mRNA levels and log transformed for statistical analysis. Normal and non-normal distributed values were tested using unpaired t-test und Mann-Whitney test, respectively, with differences p<0.05 considered significant. Results are presented as mean ± SEM, each symbol represents a single sample. (**B**) Correlations between levels of HDAC1 mRNA and FEV_1_ and the ratio FEV_1_/VC in epithelial cell biopsies from COPD patients (N = 34) and healthy controls (N = 10). Analysis was performed using Pearson correlation with differences p<0.05 considered significant. Each symbol represents a single sample.
(PDF)

Figure S3 **Correlations between levels of HDACs und DEFB1 mRNA in bronchial epithelial cell biopsies.** Correlations between transcript levels of HDAC1 to 11 mRNA and DEFB1 mRNA in epithelial cell biopsies from COPD patients (N = 34) and healthy controls (N = 10). Transcript levels of HDACs mRNA were corrected by β_2_-M mRNA levels and log transformed for statistical analysis. Normal and non-normal distributed values were tested using Pearson and Spearman correlation analysis, respectively, with differences p<0.05 considered significant. Each symbol represents a single sample.
(PDF)

Table S1 **Detailed characteristics of the study participants (additional to** **Table 1** **).** Data are presented as mean±Std (range), COPD: chronic obstructive pulmonary disease, FVC: forced vital capacity, FEV_1_: forced expiratory volume in one second, PEF: peak expiratory flow, PIF: peak inspiratory flow, VC: vital capacity, IC: inspiratory capacity, RV: residual volume, TLC: total lung capacity, ITGV: intrathoracic gas volume, SGRQ: St George's Respiratory Questionnaire, CRP: c-reactive protein, p_A_O_2_: arterial oxygen tension, p_A_CO_2_: arterial carbon dioxide tension, % of predicted value, † not determined. Stages 1 through 4 denote severity of disease according to the Deutsche Atemwegsliga and the Deutsche Gesellschaft für Pneumologie, with higher number indicating greater severity.
(PDF)
