# Supplementary figures and images for: Increased Expression of Beta-Defensin 1 (DEFB1) in Chronic Obstructive Pulmonary Disease
Source: PLoS One. 2011 Jul 19;6(7):e21898. doi: 10.1371/journal.pone.0021898 (PMC3139569; doi:10.1371/journal.pone.0021898)

**Figure S1**

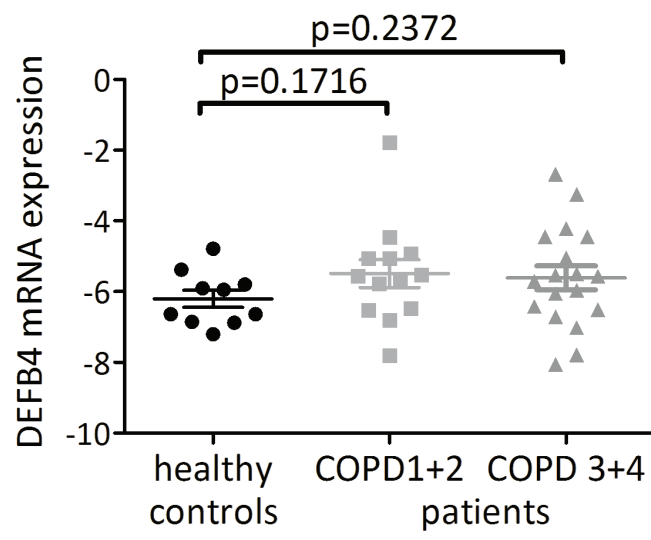

Supplement: Figure S1 — DEFB4 mRNA expression in bronchial epithelial cell biopsies from patients with COPD and healthy controls. Expression levels of DEFB4 mRNA in epithelial cell biopsies from patients with COPD (N = 34 total, N = 13 for COPD 1+2, N = 18 for COPD 3+4) and healthy controls (N = 10). Levels of DEFB4 mRNA were quantified by Real-time PCR, variations of transcript levels were corrected by β2-M mRNA levels and log transformed for statistical analysis. Differences between COPD patients (total) and healthy controls were tested using unpaired t-test. Analysis of variance of three groups was performed with the use of the One-way ANOVA test followed by Tukey's Multiple Comparison test. When the results were significant, the unpaired t-test was performed for comparison between the groups with differences p<0.05 considered significant. Results are presented as mean±SEM, each symbol represents a single sample. (PDF) [file pone.0021898.s001.pdf]

**Figure S2**

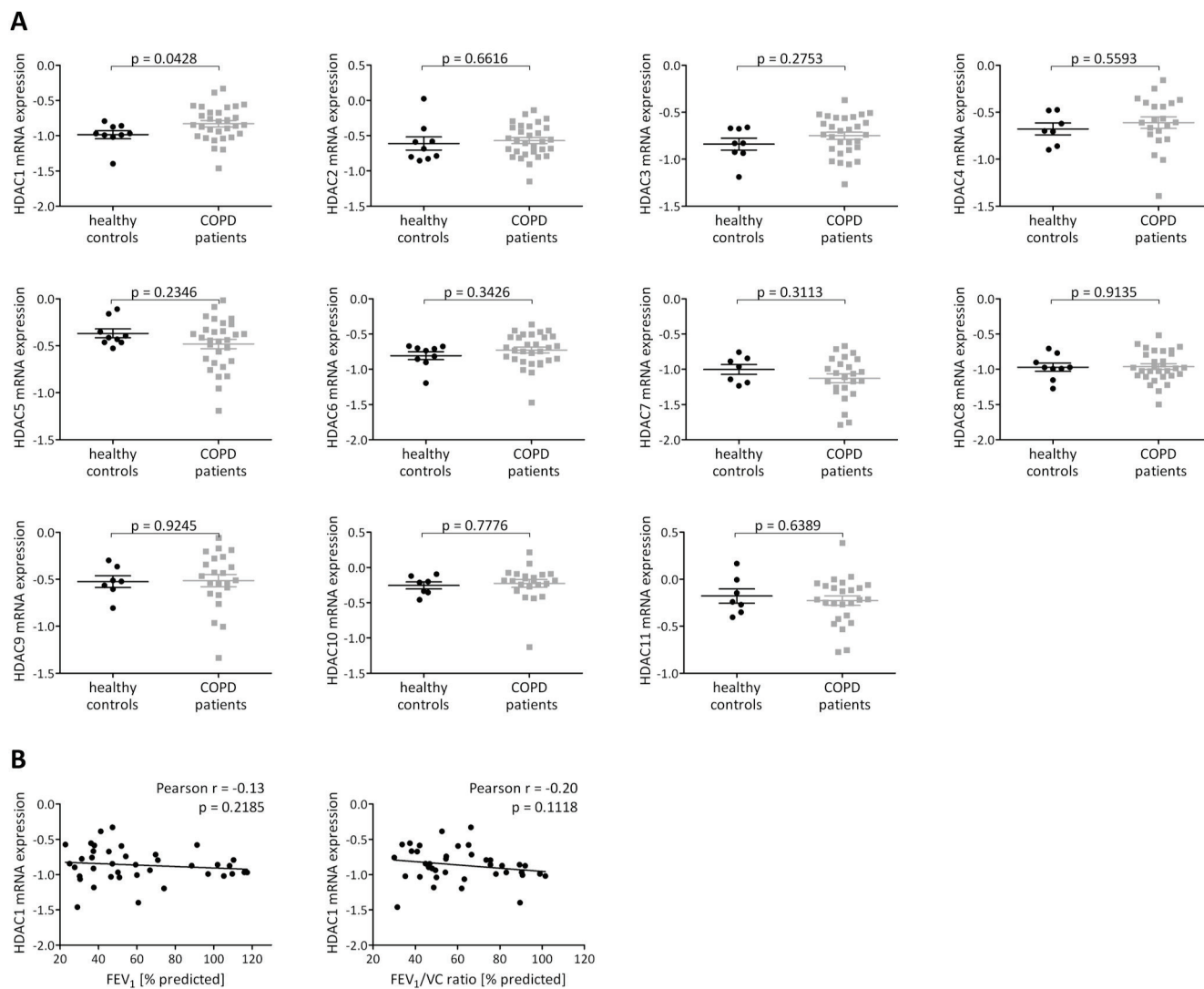

Supplement: Figure S2 — Histone deacetylase (HDAC) expression analysis in bronchial epithelial cell biopsies. (A) Expression levels of HDAC1-11 mRNA in epithelial cell biopsies from COPD patients (N = 34) and healthy controls (N = 10). Levels of HDAC mRNA were quantified by Real-time PCR, variations of transcript levels were corrected by β2-M mRNA levels and log transformed for statistical analysis. Normal and non-normal distributed values were tested using unpaired t-test und Mann-Whitney test, respectively, with differences p<0.05 considered significant. Results are presented as mean ± SEM, each symbol represents a single sample. (B) Correlations between levels of HDAC1 mRNA and FEV1 and the ratio FEV1/VC in epithelial cell biopsies from COPD patients (N = 34) and healthy controls (N = 10). Analysis was performed using Pearson correlation with differences p<0.05 considered significant. Each symbol represents a single sample. (PDF) [file pone.0021898.s002.pdf]

**Figure S3**

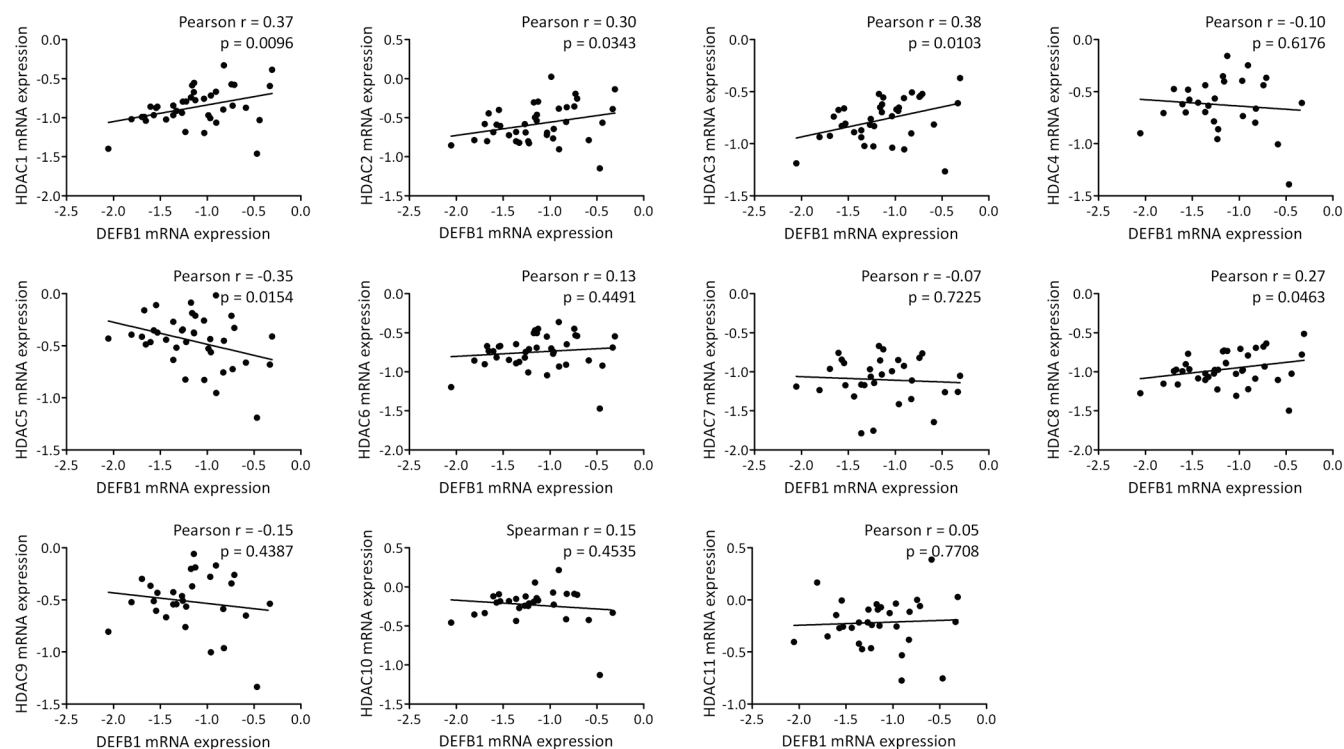

Supplement: Figure S3 — Correlations between levels of HDACs und DEFB1 mRNA in bronchial epithelial cell biopsies. Correlations between transcript levels of HDAC1 to 11 mRNA and DEFB1 mRNA in epithelial cell biopsies from COPD patients (N = 34) and healthy controls (N = 10). Transcript levels of HDACs mRNA were corrected by β2-M mRNA levels and log transformed for statistical analysis. Normal and non-normal distributed values were tested using Pearson and Spearman correlation analysis, respectively, with differences p<0.05 considered significant. Each symbol represents a single sample. (PDF) [file pone.0021898.s003.pdf]
