# Supplementary material for: Increased Expression of Beta-Defensin 1 (DEFB1) in Chronic Obstructive Pulmonary Disease
Source: PLoS One. 2011 Jul 19;6(7):e21898. doi: 10.1371/journal.pone.0021898 (PMC3139569; doi:10.1371/journal.pone.0021898)
Supplement: Table S1 — Detailed characteristics of the study participants (additional to Table 1 ). Data are presented as mean±Std (range), COPD: chronic obstructive pulmonary disease, FVC: forced vital capacity, FEV1: forced expiratory volume in one second, PEF: peak expiratory flow, PIF: peak inspiratory flow, VC: vital capacity, IC: inspiratory capacity, RV: residual volume, TLC: total lung capacity, ITGV: intrathoracic gas volume, SGRQ: St George's Respiratory Questionnaire, CRP: c-reactive protein, pAO2: arterial oxygen tension, pACO2: arterial carbon dioxide tension, % of predicted value, † not determined. Stages 1 through 4 denote severity of disease according to the Deutsche Atemwegsliga and the Deutsche Gesellschaft für Pneumologie, with higher number indicating greater severity. (PDF) [file pone.0021898.s004.pdf]

Table S1.

Characteristic	Healthy controls	COPD patients			
		Stage 1	Stage 2	Stage 3	Stage 4
Number	10	2	13	15	4
FVC [l]	4.4±1.2 (2.8-7.0)	3.0±1.5 (2.0-4.1)	3.1±0.5 (2.3-3.9)	2.3±0.6 (1.4-3.4)	1.9±0.3 (1.6-2.3)
FVC [%]	105.2±19.0 (56.6-122.9)	112.3±0.6 (111.9-112.7)	87.3±13.7 (64.9-111.8)	64.5±14.4 (41.1-81.5)	54.1±8.7 (44.2-63.9)
FEV_1 [l]	3.7±0.9 (2.5-5.2)	1.9±0.8 (1.3-2.5)	1.8±0.4 (1.1-2.6)	1.1±0.2 (0.8-1.6)	0.7±0.1 (0.7-0.9)
PEF [l/sec]	8.4±1.6 (6.7-11.0)	4.8±1.8 (3.5-6.1)	4.4±1.9 (2.0-9.1)	5.1±8.0 (1.4-33.6)	1.9±0.5 (1.3-2.4)
PEF [%]	106.6±6.8 (74.1-150.3)	76.1±3.9 (72.2-80.0)	59.4±5.9 (27.9-107.2)	42.4±4.3 (22.3-82.5)	26.2±1.7 (21.3-28.5)
MEF_{25} [l/sec]	2.0±0.7 (0.7-3.3)	0.4±0.1 (0.3-0.5)	0.4±0.2 (0.2-0.7)	0.2±0.1 (0.1-0.4)	0.2±0.1 (0.1-0.2)
MEF_{25} [%]	87.1±21.4 (52.7-115.3)	38.7±1.2 (37.8-39.5)	29.4±10.4 (16.6-49.4)	16.5±5.8 (9.2-31.2)	10.6±1.7 (8.7-12.8)
MEF_{50} [l/sec]	4.4±1.0 (2.5-6.2)	1.1±0.3 (0.9-1.3)	1.1±0.4 (0.6-2.1)	0.5±0.1 (0.3-0.7)	0.3±0.1 (0.2-0.4)
MEF_{50} [%]	90.4±15.8 (63.8-109.8)	31.6±1.5 (30.5-32.7)	27.3±8.8 (16.9-46.4)	11.2±3.2 (7.4-18.3)	7.1±1.5 (5.3-1.5)
MEF_{25-75} [l/sec]	4.0±1.1 (2.1-6.2)	1.0±0.3 (0.7-1.2)	1.0±0.4 (0.5-1.9)	0.4±0.1 (0.3-0.7)	0.3±0.1 (0.2-0.4)
MEF_{25-75} [%]	95.2±18.3 (63.7-118.1)	36.9±4.0 (34.1-39.7)	30.7±11.0 (17.8-56.6)	13.9±3.3 (9.7-21.4)	8.4±1.9 (6.5-11.0)
PIF [l/sec]	4.8±1.6 (1.6-7.1)	4.0±1.6 (2.8-5.1)	4.1±1.2 (1.9-5.9)	3.4±1.5 (1.3-7.2)	2.9±0.3 (2.5-3.1)
VC [l]	4.4±1.2 (2.7-7.0)	3.0±1.4 (2.0-4.1)	3.1±0.5 (2.2-4.0)	2.6±0.6 (1.7-3.8)	2.2±0.4 (1.8-2.6)
IC [l]	2.7±0.9 (1.8-4.6)	1.5±1.2 (0.7-2.4)	1.9±0.5 (0.9-2.6)	1.4±0.4 (0.6-1.9)	1.1±0.3 (0.9-1.4)
IC [%]	91.6±20.3 (47.6-113.3)	67.6±27.4 (48.2-86.9)	70.0±17.0 (43.8-95.6)	50.8±15.1 (16.5-84.8)	46.1±17.0 (26.7-67.4)

Characteristic	Healthy controls	COPD patients			
		**Stage 1**	**Stage 2**	**Stage 3**	**Stage 4**
RV [l]	1.6±0.8 (0.6-3.1)	1.9±0.7 (1.5-2.4)	3.2±0.8 (1.4-5.0)	4.1±1.3 (2.0-6.8)	5.0±0.6 (4.3-5.7)
RV [%]	98.1±38.3 (37.0-144.1)	83.6±16.5 (72.1-95.4)	136.5±39.0 (50.6-183.0)	175.4±59.2 (76.4-257.2)	247.5±28.6 (224.5-285.5)
TLC [l]	6.0±1.8 (3.8-10.1)	5.0±2.1 (3.5-6.4)	6.3±1.1 (4.1-8.4)	6.6±1.2 (4.9-8.8)	7.2±1.0 (6.1-8.4)
TLC [%]	101.5±19.3 (53.5-121.2)	91.1±13.1 (81.8-100.3)	102.2±20.6 (56.7-134.3)	107.5±22.9 (70.8-142.4)	126.2±16.2 (110.9-149.1)
ITGV [l]	3.3±1.0 (2.0-5.5)	3.4±0.9 (2.8-4.1)	4.4±1.0 (2.6-7.1)	5.3±1.0 (3.7-7.2)	6.1±0.9 (5.2-7.4)
ITGV [%]	110.9±22.1 (60.5-145.0)	114.1±3.4 (111.7-116.5)	134.7±32.7 (68.1-192.3)	160.8±35.0 (102.5-210.1)	198.6±13.6 (186.7-217.9)
FEV_1/FVC [l]	0.9±0.1 (0.7-0.9)	0.6±0.04 (0.6-0.7)	0.6±0.1 (0.4-0.8)	0.5±0.1 (0.4-0.6)	0.4±0.01 (0.39-0.42)
IC/TLC [l]	0.4±0.03 (0.4-0.5)	0.3±0.1 (0.2-0.4)	0.3±0.1 (0.2-0.4)	0.2±0.1 (0.1-0.3)	0.2±0.04 (0.1-0.2)
RV/TLC [l]	0.3±0.1 (0.1-0.4)	0.4±0.04 (0.37-0.42)	0.5±0.1 (0.4-0.6)	0.6±0.1 (0.4-0.8)	0.7±0.02 (0.68-0.73)
SGRQ	1.28±1.25 (0.0-3.0)	†	54.8±24.4 (28.8-90.3)	68.7±11.5 (52.0-77.8)	61.0±1.0 (52.5-71.9)
CRP [mg/dl]	†	0.24±0.2 (0.1-0.4)	1.4±1.6 (0.2-5.8)	0.9±1.4 (0.1-5.5)	0.51±0.53 (0.1-1.3)
p_AO_2 [mm Hg]	84.8±4.8 (77.1-91.8)	65.4±8.0 (59.7-71.0)	71.1±10.5 (49.9-84.8)	63.3±9.1 (46.3-84.6)	64.9±12.5 (47.4-74.5)
p_ACO2 [mm Hg]	40.2±4.4 (33.6-46.1)	35.0±4.5 (31.8-38.1)	40.4±5.0 (32.9-52.3)	41.7±5.1 (35.2-53.0)	48.3±7.0 (41.2-57.7)
